# Utility of decision tools for assessing plant health risks from management strategies in natural environments

**DOI:** 10.1002/ece3.11308

**Published:** 2024-05-02

**Authors:** Flora Donald, Carrie Hedges, Bethan V. Purse, Nik J. Cunniffe, Sarah Green, Festus A. Asaaga

**Affiliations:** ^1^ UK Centre for Ecology and Hydrology Wallingford Oxfordshire UK; ^2^ Department of Plant Sciences University of Cambridge Cambridge UK; ^3^ Forest Research, Northern Research Station Roslin Midlothian UK; ^4^ Institute of Science and Environment University of Cumbria Ambleside UK; ^5^ Unit 6 Cumbria Woodlands Kendal Cumbria UK

**Keywords:** biosecurity, co‐production, decision tools, habitat restoration, plant health, policy process

## Abstract

Increased imports of plants and timber through global trade networks provide frequent opportunities for the introduction of novel plant pathogens that can cross‐over from commercial to natural environments, threatening native species and ecosystem functioning. Prevention or management of such outbreaks relies on a diversity of cross‐sectoral stakeholders acting along the invasion pathway. Yet, guidelines are often only produced for a small number of stakeholders, missing opportunities to consider ways to control outbreaks in other parts of the pathway. We used the infection of common juniper with the invasive pathogen *Phytophthora austrocedri* as a case study to explore the utility of decision tools for managing outbreaks of plant pathogens in the wider environment. We invited stakeholders who manage or monitor juniper populations or supply plants or management advice to participate in a survey exploring their awareness of, and ability to use, an existing decision tree produced by a coalition of statutory agencies augmented with new distribution maps designed by the authors. Awareness of the decision tree was low across all stakeholder groups including those planting juniper for restoration purposes. Stakeholders requested that decision tools contain greater detail about environmental conditions that increase host vulnerability to the pathogen, and clearer examples of when management practices implicated in pathogen introduction or spread should not be adopted. The results demonstrate the need to set clear objectives for the purpose of decision tools and to frame and co‐produce them with many different stakeholders, including overlooked groups, such as growers and advisory agents, to improve management of pathogens in the wider environment.

## Graphical abstract

Decision tools are often created by policymakers to help practitioners reduce the risk of introduction and spread of plant pests and diseases in natural environments. We show practitioners are eager to risk assess management actions in the context of plant health but decision tools must be designed with clear aims, broad stakeholder mapping, and locally relevant data. Stakeholders articulated the need for tools that better support decision‐making regarding the risk of introducing pathogens through restoration plantings.

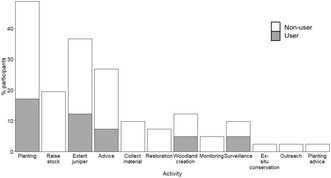

## 1 INTRODUCTION

As the scale of global tree disease epidemics increases, so too does the scope and number of actors involved (Marzano et al., 2016). Almost one‐fifth of the Earth's surface is estimated as at risk of plant and animal invasions (Intergovernmental Science‐Policy Platform on Biodiversity and Ecosystem Services [IPBES], 2019), with increasing numbers of plant pest and pathogen introductions, resulting from increased global trade in horticultural plants, crops and timber (Brasier, 2008; Chapman et al., 2016). In the UK, approximately five new biotic threats are added to the national Plant Health Risk Register every month, of which 30% are identified as capable of infesting or infecting trees (Department for Environment Food and Rural Affairs [DEFRA], 2018). Such outbreaks constitute severe economic losses (Hill et al., 2019), contribute to serious biodiversity loss (IPBES, 2019) and result in detrimental changes to ecosystem functioning (Boyd et al., 2013) with adverse consequences for human health (Maier et al., 2003) and livelihoods (DEFRA, 2018).

Many pests and pathogens are introduced by anthropogenic behaviours (Brasier, 2008). In a plant health context, individuals or organisations can be defined as stakeholders when they can affect and/or are affected by pest or disease outbreaks (after Freeman, 1984). Under this definition, responsibility for plant health in the wider environment extends not only to land owners/managers responsible for managing habitats but also to (i) agents or funders who can influence whether biosecurity is prioritised and stipulate design principles that determine how quickly pests or diseases might spread once introduced to a site, (ii) growers whose practices can determine if a pest or disease is introduced to a site with new plants and (iii) contractors and recreational users who may transport pest and disease propagules over varying distances on vehicles, machinery or footwear. Success in changing such stakeholder actions to prevent or mitigate the introduction and spread of plant pests and diseases depends, then, on the translation of knowledge into practice across different sectors and spatial scales. New plant pest and disease knowledge is regularly generated by researchers tracking distributions, assessing new threats, and developing models to predict spread under climatic changes (Kleczkowski et al., 2020) or alternative management scenarios (Bate et al., 2016; Cunniffe et al., 2016). Yet, communicating these inferences to stakeholders is rarely an explicit focus of such studies (Cunniffe et al., 2015; Gaydos et al., 2021). Even when stakeholders are involved in plant health research this is often only at the implementation stage (e.g. outbreak monitoring) rather than to frame research questions or policy design (Dandy et al., 2017; Reed, 2008).

Though methods used to prevent or contain pest or pathogen, outbreaks will vary with species, landscape, and spread pathway, stakeholder perceptions of research credibility, relevance, and legitimacy (CRELE) are critical factors that determine how successfully research is translated into management action. Relevance is most important and can be further disaggregated into ACTA attributes defined as *applicability*, *comprehensiveness*, *timing*, and *accessibility* (Dunn & Laing, 2017). *Applicability* is defined as the specificity of evidence to the problem and crucially its useability for solving it. For example, if there is a mismatch between the data required by a research solution and the data collected by stakeholders, the solution is unlikely to be adopted by the intended audience (Dunn & Laing, 2017; Jones & Kleczkowski, 2020). *Timing* describes the alignment of research outputs with a window of opportunity for stakeholder action, for example, is knowledge transferred in time for symptoms to be visible on seasonal hosts or for annual resource allocations to be altered (Cook et al., 2017)? *Comprehensiveness* requires that research is contextualised with other key considerations valued by stakeholders (e.g. socio‐economic impacts) to make changes in practice easier to adopt because the impacts on other business areas are clear. Finally, the *accessibility* attribute of ACTA focuses on how and where evidence is communicated, for example, clear, jargon‐free messaging in a freely available, trusted location. The small number of studies that have explored how plant health research is disseminated to stakeholders found academic research is highly trusted but difficult for practitioners to find and access (Creissen et al., 2019; Marzano et al., 2016) and stakeholder engagement was more effective when employing interactive learning (White et al., 2018).

Our study used the infection of UK populations of common juniper (*Juniperus communis* L.) with the introduced oomycete pathogen *Phytophthora austrocedri* Gresl. & E. M. Hansen as an example to understand barriers to plant health guidance application. We chose *P. austrocedri* as an example of an ongoing plant disease outbreak that requires action from a wide variety of stakeholders based in multiple sectors for successful control. The pathogen primarily infects juniper via the roots, dispersing as short‐lived zoospores in soil water (Green et al., 2015). It is frequently found in plant nurseries and disturbed soils in the wider environment, it is also likely spread by movements of infected soil by animals and vehicles or associated with ‘plants for planting’ (Green et al., 2021; Landa et al., 2021; Riddell et al., 2019). Infection generally spreads from the roots to cause necrotic lesions that girdle the phloem and cause extensive tree mortality in juniper populations right across Scotland and England, including in the most significant UK refugia in the Cairngorms and the Lake District (Green et al., 2015). Information about symptoms caused by the pathogen, its dispersal pathways and impacts on juniper is available on websites catering to a variety of audiences including land managers, members of environmental charities and citizen scientists (Forest Research, Woodland Trust, Observatree and the Arboricultural Association).The pest risk analysis for *P. austrocedri* is available in summary and detailed form on the UK Plant Health Risk Register (DEFRA, 2015).

Public and private land managers, conservation organisations, independent consultants, commercial growers, gin producers and environmental regulators all have an interest in, and some ability to, maintain disease‐free populations. Prior to disease detection, UK juniper populations were already in decline and a proliferation of technical guides to support juniper conservation using techniques such as grazing regulation, scrub removal and seed scrapes were published from the early 2000s onwards by the GB forestry regulator and the well‐known Plantlife charity (Broome, 2003; Forestry Commission, 2013; McCartan & Gosling, 2013; Plantlife International, 2007; Wilkins & Duckworth, 2011). Plantlife organised nationwide citizen science surveys of juniper populations in 2004–2005 and 2013–2015 and published compendiums of information about juniper that included management advice to update their technical guides (Plantlife, 2015; Ward & Shellswell, 2017). Considerable effort and expense was exerted by statutory agencies, conservation charities, utility companies, community groups, national parks and private individuals to conserve juniper populations in the wider environment (Ward & Shellswell, 2017) but the action most commonly taken to improve the age structure, regenerative capacity and extent of native habitats that included juniper was to bring in new juniper plants to supplement those already present on site (Donald et al., 2021). It is likely that some of these planting events introduced *P. austrocedri* to juniper populations. Statutory action is currently taken to prevent the movement of *P. austrocedri* between plant nurseries but not in the wider environment where no remedial options exist to eradicate infection (DEFRA, 2017).

The UK Plant Health Risk Group commissioned writing of juniper management guidelines to bring together information about managing juniper populations in the wider environment with information about managing *P. austrocedri* (Barbrook, 2024, pers. comm). The guidelines were written by technical specialists based in agencies responsible for plant and forest health in GB, for an audience of land managers, conservation organisations and nurseries, to help them identify risks and implement good practice and resulting in sustainable juniper populations (DEFRA, 2017). A decision tree was included to guide land managers through a risk assessment flow chart of yes/no questions that examine the vulnerability of a particular juniper population to extinction because of its size, structure, site conditions or known presence of *P. austrocedri* (DEFRA, 2017). Outcomes reached via the decision tree are statements that the site is unsuitable for planting, requires biosecurity actions, or is suitable for planting with expert advice and accompanying biosecurity. Once finalised, the guidelines were published by the UK Government Department for Environment, Food and Rural Affairs (DEFRA), hosted as a 30 page, free to download, document on the Scottish Plant Health Centre website and signposted to from the Defra UK Plant Health Information Portal. The guidelines were sent to all agencies involved in drafting them for wider distribution and shared by agency staff in response to contact with land managers, nurseries or gin manufacturers managing or trading juniper (Barbrook, 2024, pers. comm).

We conducted a multistakeholder survey across sectors involved in juniper management asking the over‐arching question: “to what extent are decision tools currently used to aid risk assessment of the *P. austrocedri* disease threat in relation to juniper populations and how could they be improved?”. Survey responses were analysed to identify stakeholder needs to improve the relevance of the juniper management guidelines, co‐design our planned *P. austrocedr*i risk model and inform dissemination of the results to improve management of *P. austrocedri* in the UK. However, the responses reflect stakeholder perceptions and barriers to decision tool use and guideline application, particularly relevant to assessing plant health risks associated with habitat restoration. We use these to design a framework for iterative design of decision tools, using juniper as an example that incorporates ACTA principles to improve guideline relevance, uptake and ultimately impact in reducing pest or pathogen establishment and spread.

## 2 METHODS

### 2.1 Ethics statement

Participation in the stakeholder survey was entirely voluntary. Before starting the survey, participants were asked to consent to the ethics statement outlining their confidentiality, right to withdraw, and request the removal of responses. Prior to the thematic analysis, responses were randomly ordered and all identifying information was removed to ensure that themes were analysed without any pre‐disposing information. Stakeholder type was then re‐introduced to permit analysis within these categories. All participants provided written consent for their data to be analysed and reported on in a journal article.

### 2.2 Conceptual stakeholder categorisation

We restricted participation to stakeholders who perform a role connected with juniper management as the target audience for the decision tools. Within organisations, stakeholders can take role‐specific approaches to risk assessment that may vary with spatial scale (e.g. local or national focus) and stage of invasion (e.g. prevention vs management following invasion). We therefore requested that participants respond based on their own role and this description was used to assign each participant to a stakeholder type (Table 1), allowing us to explore decision tool preferences and usage barriers within these groups. Illustrative quotes are reported in this study using the random number of the participant who offered it in the assigned stakeholder group (e.g. Agent 1).

**TABLE 1:** Description of four stakeholder types involved in risk assessment and decision‐making about populations of common juniper in the UK wider environment.

Stakeholder type	Description
Agents	private or charitable sector employees provide independent (paid) advice devise management plans recommend biosecurity practices (e.g. vehicle washing, footpath diversions, sources of disease‐free plants) liaise with stakeholders are not responsible for implementing management
Assessors	public sector employees provide non‐commercial management advice, for example, woodland, species, or biodiversity advisers within statutory agencies perform a regulatory function, for example, comment on planning applications, provide protected area consents for management conduct monitoring, for example, disease surveillance evaluate funding applications pertinent to juniper restoration or creation may advise, recommend, or evaluate biosecurity practices and could make biosecurity conditional for grant or contract awards
Growers	Private sector employees Supply juniper commercially by either raising stock themselves or importing plants Raise and maintain disease‐free stock
Managers	public, private, or charitable sector employees involved in day‐to‐day management of juniper populations for any purpose, for example, conservation, gin production could include the landowners themselves, tenants, or agencies who manage land on behalf of the landowner may grow juniper themselves and trade plants non‐commercially implement and enforce on‐site biosecurity practices, for example, restricting movements between diseased and disease‐free zones, quarantining planting stock

### 2.3 Survey design

A self‐completion questionnaire was designed consisting of 21 open and closed format questions of which 13 were mandatory (Supplementary Information S1). The first question asked stakeholders to explain their experience and role to aid identification of stakeholder type, followed by two yes/no questions asking if their role involved management of juniper and/or *P. austrocedri* and supplementary juniper planting. The survey was then presented in two main sections: (i) six questions pertaining to the awareness and use of the decision tree presented in the juniper management guidelines (DEFRA, 2017) and (ii) nine questions about the sources and utility of spatial information (distribution maps) followed by three questions about the expected importance of potential infection risk factors. We created and presented two, UK‐wide, interactive maps in this section. The first map displayed the 2 km resolution distribution of native juniper (1990–2020) and 1 km positive detections of *P. austrocedri*. The second map overlaid the first with 2 km resolution juniper planting events conducted 1960–1979, 1980–1999, 2000–2009 or 2010–2020. Maps were created in R v.3.6.2. (R Core Team, 2019) using the datasets compiled in Donald et al. (2021) and were presented in an R Shiny app (Chang et al., 2021) using the leaflet package (Cheng et al., 2021) that allowed participants to zoom in on locations of interest against a simple topographic backdrop. The maps are reproduced in Supplementary Information S2. Stakeholders were asked to pick five of 13 abiotic, and five of 8 biotic, proposed risk factors and to rank them according to importance (5 = most important, 1 = least important). The perceived importance of potential infection risk factors was then calculated as the sum of the ranked scores (1–5) assigned to each risk factor over all participants.

Relevant stakeholders were identified by pooling our own knowledge of individuals and organisations associated with juniper in any capacity and sector we suspected retained privileged information. We initially e‐mailed the survey to a pilot sample of 13 stakeholders to check that the questions were easy to interpret and addressed the areas of research interest. This was evident from the three completed surveys received in response and no modifications were made to the survey before wider circulation. The survey was then e‐mailed to 90 additional named individuals who were requested to complete the survey within a 2‐week period in October 2020. Within the survey form, recipients were asked to suggest contacts in their network involved in “managing, growing, advising, surveying or making decisions about juniper populations” who we could invite to participate. Seven individuals directly forwarded the survey to their network whilst a further 18 individuals were recommended as contacts, 12 of whom we had already contacted. This suggests that our stakeholder mapping identified many of the influential actors. In total, we distributed the survey to 109 individuals (not including those forwarded by recipients) and received 41 completed surveys including the pilot responses. Despite the small sample size, we believe it is defensible to present the results because the stakeholder mapping exercise was thorough and suggests a response rate of 38%, which is greater than the response rates of 29%–32% reported by forestry studies with broader remits (Marzano et al., 2016).

A short section at the end of the survey asked participants to provide their job title and a description of their role or specialisation. This information was used to assign their responses to a stakeholder type (agent, assessor, grower, manager; Table 1). Participants were well distributed across categories, with managers constituting the largest group of stakeholders (*n* = 15, 37%), followed by agents (*n* = 11, 27%), assessors (*n* = 9, 22%) and growers (*n* = 6, 15%). A greater percentage of growers responded to the survey (60% of those contacted) compared with the other stakeholder types (32% of assessors, 33% of managers and 39% of agents).

Survey questions addressing the main subject areas were grouped together and the corresponding responses were analysed using an open, line‐by‐line coding strategy where keywords or important phrases were identified and organised into clusters with shared meaning (Braun & Clarke, 2006). Theme frequencies were explored and presented as the number and percentage of responses in total and/or according to stakeholder type. Statistical differences in responses between stakeholder types or countries were assessed using Fisher's exact test and the resulting *p*‐values were adjusted using the Holm‐Bonferroni method to control for multiple comparisons, all implemented using the stats package in R v.3.6.2 (R Core Team, 2019). Three main themes were identified using the theme frequencies and keyword clusters (Table 2).

**TABLE 2:** Summary of main and sub‐themes identified from all survey responses.

Main theme	Sub‐themes
Juniper management guidelines aren't reaching the intended audience	Awareness was low across participants: from all stakeholder groups who supply juniper or juniper planting advice who manage existing juniper populations, and new woodland creation schemes
The decision tree is useful but requires changes to improve relevance	the purpose of the tree was unclear—is it only relevant for decisions involving juniper planting? participants preferred using the tree in conjunction with local distribution maps juniper site‐level suitability and vulnerability checklists were the highest rated components of the tree Recommendations lacked detail required for application
Assessing risks associated with planting juniper was of key importance to stakeholders	Over 50% of participants were involved in planting juniper Participants ranked juniper planting as a major biotic risk factor for introducing disease More decision tree users accessed it to find planting alternatives than to conduct juniper planting Managers and assessors stated they were likely to use the tree to assess future planting decisions Planting recommendations were variously viewed as ambiguous, too risk averse or too relaxed

## 3 RESULTS

### 3.1 Low awareness of guidelines

Three years following the publication of the decision tree on p.2 of the juniper management guidelines (DEFRA, 2017), 71% of survey participants who conducted a role connected with juniper management reported they did not use it. A total of 65% of participants explicitly stated they were unaware of the guidelines, including Grower 2:

I was unaware of it [the decision tree], despite having done a reasonable amount of reading on the subject.

**FIGURE 1:**
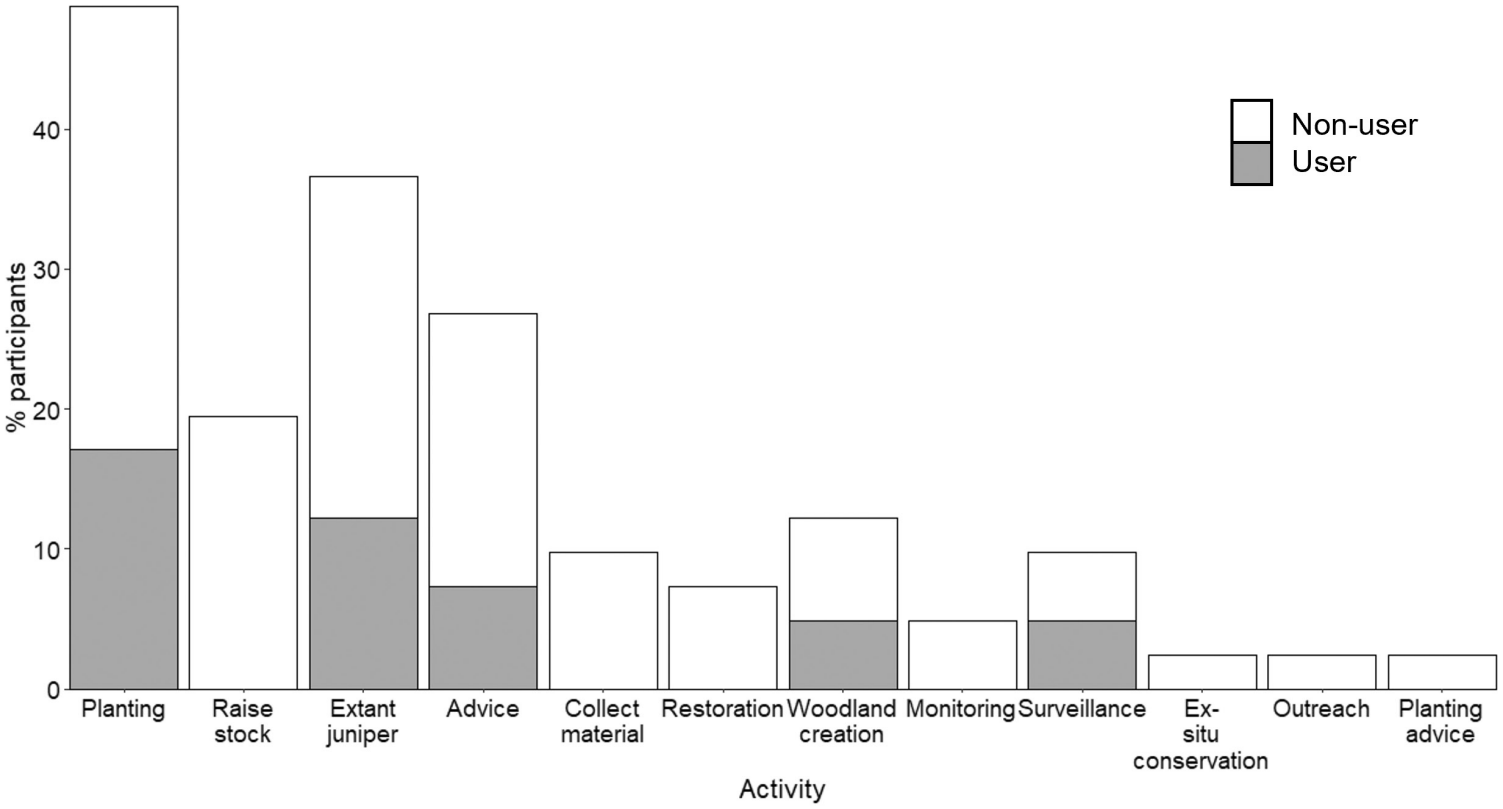
Percentage of participants describing activities as part of their current role (sum of grey and white bars), differentiated between participants who used (grey bars), and did not use (white bars), the decision tree, ordered by the percentage of non‐users. General “advice” was categorised separately from responses detailing delivery of “planting advice”; “monitoring” of existing juniper populations was categorised separately from plant health monitoring defined as “surveillance”; management of “extant juniper” populations was distinguished from “ex situ conservation” of juniper. Longer descriptions of each activity and how these relate to the stakeholder types are given in Supplementary Information S3.

Awareness was poor across all stakeholder groups. Assessors showed higher awareness (56%) compared with managers (40%), agents (9%) and growers (0%) but these differences were not statistically significant (Holm‐Bonferroni corrected *p*‐values = .24). There was no awareness of the decision tree amongst participants whose stated role involved collecting or raising planting stock, ex situ conservation, planting advice or outreach, and no awareness in large percentages of those involved in woodland creation (60%), managing (67%) or offering advice (72%) about existing juniper populations (Figure 1).

### 3.2 Relevance of juniper decision tools

Participants exhibited a strong preference to use both the decision tree and interactive maps (61%) compared with the maps (22%) or decision tree (12%) alone (Supplementary Information S3). Both positive and negative feedbacks were obtained from participants who had not previously encountered the decision tree:

I wouldn't consider planting juniper unless I had gone through a similar process of risk assessment to this decision tree. (Grower 6), versus

It is an extra layer to management decisions which can make it a diversion. Ignores experience. (Agent 1)

When asked to identify the most useful sections of the decision tree (irrespective of current use) 22% of participants said all of it was useful but 17% specifically identified the juniper site suitability checklist (Supplementary Information S3), writing for example:

Is the site suitable to that species. After this can we control human and environmental impacts on the site. (Manager 3)

and 12% referred to the checklist that assesses the vulnerability of juniper populations by virtue of their size and potential for natural regeneration:

The levels of vulnerability are useful to consider how best to deal with existing populations, they give cause to stop and think. (Grower 2)

Three participants (7%) noted the decision tree was useful to provide an architecture for risk assessment (i.e. the process is intrinsically useful) and three participants (7%) suggested they would use it to assess the need or potential longevity of planting. Agent 6 noted that the decision tree raised their awareness of biosecurity:

I haven't in the past considered too strongly biosecurity issues but would do so now.

The scale (national, regional, or local) of map preferred by participants depended on their geographical remit but most requested local maps (39%) or local maps that could be contextualised by national scale maps (27%) (Supplementary Information S3). The most popular uses of interactive maps were to assess the risk of *P. austrocedri* infection (27%), to inform management decisions (15%), assess the site suitability for supplementary planting (12%) or to choose a source of donor material (10%). The financial benefits of accurate distribution maps were discussed by Agent 4 as “good evidence” to support grant applications, and by Manager 14 who identified planting juniper in infected areas would “result in more expenditure to the client.”

Confusion about the purpose of the decision tree was apparent at several points in survey. Lack of involvement in juniper planting (13%) or conducting planting prior to guideline publication (10%) were cited as reasons for not using the decision tree, suggesting these participants thought it only applied to planting decisions. Manager 9 wrote:

There is a lot of information within the decision tree that does not relate to planting – it is more about an overall management approach for *P. austrocedri*.

Twelve additional barriers to using the decision tree were identified from the responses, half of which were described by ≥10% of participants (Table 3). One manager wrote they were unaware of *P. austrocedri* whilst two agents conflated risk factors for *P. austrocedri* with those for *P. ramorum* presence of rhododendron hosts (Purse et al., 2013) and prevailing winds (Rizzo et al., 2005) demonstrating a lack of pathogen specific knowledge. Lack of knowledge about *P. austrocedri* identification and distribution, biosecurity measures, sourcing considerations, and where to seek advice to limit spread, featured as recurrent perceived problems across the survey (Table 3). Some of these topics (biosecurity and plant/seed sourcing) are included in a wider document but not signposted from the decision tree (DEFRA, 2017). Several participants suggested recommendations within the decision tree were complicated, ambiguous, or too discretionary:

The questions are open to interpretation and professional judgement. (Assessor 2)

**TABLE 3:** Number (*n* = 41) and percentage (in brackets) of participants who identified similar themes as barriers to using the decision tree with an example quote summarising the theme.

Barrier	*n*	Exemplar quote
Lack of diagnostic information	5 (12%)	“I do not know what the signs are of the juniper disease.” (Manager 14)
Inappropriate planting scenarios are not made explicit	5 (12%)	“It's clear in the red boxes that planting is not recommended, but not clear at boxes 4 and 5. Should they have red outlines, or is there ambiguity in advice here?” (Manager 7)
Uncertain where to seek expert advice	5 (12%)	“Seek expert advice (not sure who to contact)” (Agent 2)
Infection “proximity” is poorly defined	5 (12%)	“Unfortunately there are no parameters for “proximity of any known juniper infection” … and no guidance about how far from infected juniper is safe to plant.” (Assessor 6)
Unclear definition of “water catchment”	4 (10%)	“at 2 does river catchment area mean the entire catchment? … It's a big area to rule out planting anywhere.” (Manager 10)
Insufficient detail to assess site suitability	4 (10%)	“It would be useful to include a quick reference for suitable ranges for each factor that needs to be assessed for suitability.” (Manager 9)
Local disease distribution information is inaccessible	3 (7%)	“Forest Research map of confirmed locations are insufficiently detailed to confirm whether P. austrocedri is in a catchment.” (Agent 2)
Ambiguous recommendations	3 (7%)	“I think I need to be talked through the decision tree to really understand the final recommendations.” (Assessor 3)
Biosecurity actions are not clearly articulated	2 (5%)	“worth pointing people towards what “high‐risk biosecurity” measures involve? I don't think this is spelled out in the guidance document itself.” (Assessor 4)
No advice about sourcing of planting material	2 (5%)	“this document completely misses out a section on verification and disease risk reduction in seed collecting and suitability of potential planting stock.” (Grower 4)
Recommendations contradict protected area aims	1 (2%)	“The decision tree does not quite reflect the position of the SAC designation. Although we have a large population the age structure means we have a high proportion of old juniper with little viable seed germination, therefore planting is undertaken.” (Manager 2)
No emphasis on population sustainability requiring both male and female trees	1 (2%)	“It might be useful to explain that juniper is dioecious, and therefore it will be important to make sure that both sexes are present, and only look for seeds on female trees.” (Assessor 8)

The lack of detail relating to “safe” distances from the nearest *P. austrocedri* outbreak, microsite conditions preferred by juniper, and natural regeneration requiring male and female trees were also identified as omissions limiting implementation (Table 3).

To understand how awareness of the decision tools could be raised, participants were asked to identify sources of juniper management information they currently access. A handful of sources were accessed by small numbers of participants, comprising a combination of private and publicly accessible resources, showing no single repository is used to access information about juniper or *P. austrocedri* (Supplementary Information S3).

Preferred locations to host decision tools included websites already used by participants to source information (9%) (Supplementary Information S3) or existing land management mapping software (12%) that would allow users to directly upload data via a web interface or app (15%). Participants stated map provision would support rather than replace site visits unless *P. austrocedri* presence was shown at the specified location (71%). However, the time‐consuming nature and cost–benefit imbalance of maintaining highly accurate distribution maps, potential complacency resulting from outdated or coarse‐scale information and the need for funding continuity, often hard to obtain for ongoing data collection projects, were highlighted as disadvantages to providing interactive maps. Concern was also raised that maps would have limited use unless widespread testing for *P. austrocedri* is undertaken, and multiple organisations work together to contain spread.

### 3.3 Risk assessment of juniper planting in relation to disease

**FIGURE 2:**
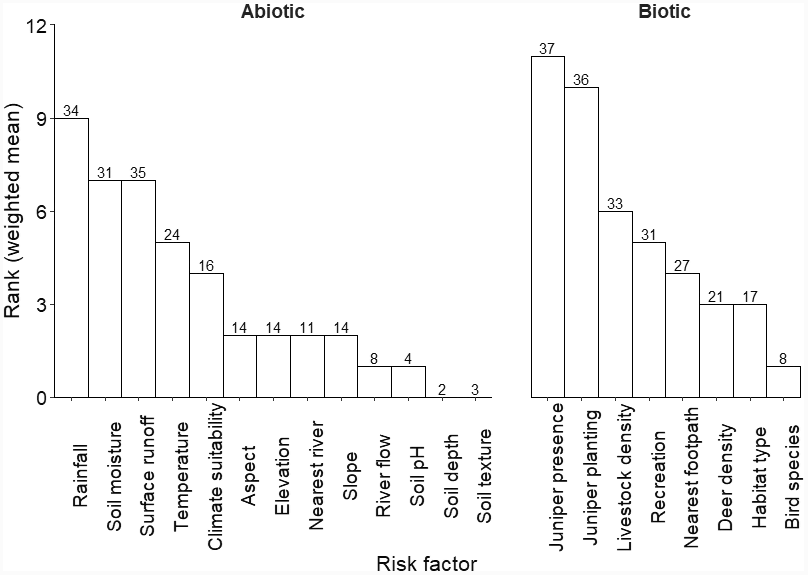
Predicted rank importance of abiotic (L) and biotic (R) risk factors proposed to drive *P. austrocedri* outbreaks in UK juniper populations. The number of votes given to each risk factor is displayed above each bar.

When participants were asked to rank abiotic and biotic factors most likely to drive outbreaks of *P. austrocedri*, four participants (10%) highlighted that their responses were based on limited knowledge. Though all proposed risk factors were selected at least once, a consensus emerged that water availability—characterised as rainfall, soil moisture or surface runoff—would be the most important abiotic risk factor (Figure 2). Juniper planting was the most important biotic risk factor after juniper presence, followed by livestock density and recreation (Figure 2). A similar percentage (~60%) of participants who did and did not plant juniper ranked “juniper planting” as the first or second most important biotic risk factor for disease. Importance rankings attributed to juniper planting did not differ statistically between stakeholder types (Supplementary Information S3).

That juniper planting was highlighted so strongly as a risk factor driving *P. austrocedri* outbreaks is interesting given 56% of participants stated they were involved in planting juniper, 70% (16/23) of whom did not access the guidelines to do so (Figure 1). The 12 current users (29% of participants) of the decision tree stated their purpose for consulting it was to assess the risk of planting (50%) but more used it for knowledge exchange (33%), to raise awareness about biosecurity (25%) and/or to find planting alternatives (25%) than to actively conduct planting (17%).

Use of the decision tree to risk assess juniper planting decisions was a consistent theme returned from all sections of the survey. When asked directly how likely they were to use the decision tree to assess the suitability of planting juniper at a proposed location, 26 participants (63%) said they were likely or very likely to. Managers were most likely to state they would use the tool for this purpose (80%) followed by assessors (78%) and lower proportions of agents (45%) and growers (33%). As detailed in the previous section, uses identified for the decision tree and interactive maps included for planting decisions (e.g. assessing the need and longevity of planting, Supplementary Information S3) and key usage barriers included ambiguity over inappropriate planting scenarios and lack of advice regarding how to source biosecure planting material (Table 3).

Manager 9 thought the decision tree presented:

clear guidance that planting should not be undertaken on sites where *P. austrocedri* is present.

Assessor 8 used the decision tree for this purpose, that is:

… helping people through the process of accepting that they might not need to plant, even though they really want to.

By contrast, Assessors 5 and 6 suggested planting was not discouraged strongly enough:

The tree … does not explicitly oppose planting. The boxes also mention grant‐aid, which suggests that planting is acceptable. If planting risks bringing in *Phytophthora* to a juniper site then perhaps no juniper should be planted in any existing juniper site? (Assessor 5)

Opposing views also emerged concerning the necessity to plant juniper to safeguard populations versus the risk of inadvertently introducing *P. austrocedri* on supplementary material, as illustrated by these two opposite positions:

I believe that most, if not all, tree nurseries are contaminated with *Phytophthora*… A planting scheme close to a juniper site could still spread *P. austrocedri* even if juniper was not planted… (Assessor 5)

It *[the decision tree]* may lead to suitable sites not being planted with juniper due to potential risks and possibly the long term decline in juniper populations across the areas that are most suitable for juniper scrub. (Manager 11)

The second view was expressed by two other participants who wrote that the decision tree could lead to risk averse decisions, resulting in worse outcomes for juniper by not replacing stands failing to naturally regenerate, ruling out too large an area as unsuitable for planting, or decision tree complexity leading to management inaction.

## 4 DISCUSSION

The proliferation of decision support, risk prioritisation and multi‐criteria analysis tools designed to help practitioners change or optimise plant health management is set to continue (Barwell et al., 2022; Jones et al., 2021). Involving users in the design, evaluation and re‐issue of such tools remains critically important, then, to ensure tools are relevant to the management context and key actors and contribute to improved outcomes. Our case study results support the use of the ACTA principles to consider key barriers and solutions to co‐producing decision tools with stakeholders.

Low awareness (29% of 41 participants) of the decision tree in the juniper management guidelines (DEFRA, 2017) across all stakeholder groups demonstrated its limited *accessibility* to the intended audience of land managers, conservation organisations and nurseries. This was the main barrier to its application but the clarity of recommendations also limited its accessibility. When stakeholders were aware of the decision tree, they used it to raise land manager awareness of *P. austrocedri* and advocate disease management practices, showing clear benefits of disseminating the tool more widely. More generally, 95% of participants identified ways the decision tools we presented would inform risk assessment and decision‐making within their role. A survey of UK practitioners involved in habitat creation or restoration found 51% did not or did not know if they had a project risk assessment for plant pests (Mitchell, 2023). This illustrates a very practical need (*timing*) for decision tools that can help raise awareness, assessment and implementation of good biosecurity practices in this space.

Participants in our survey identified multiple ways to improve the *applicability* of the decision tree and maps to their work including provision of locally detailed pathogen distribution information. Records of invasive pathogens are scarce across the globe and where distributions are monitored provide powerful information used for horizon‐scanning, disease prevention and control (Barwell et al., 2021; Bebber et al., 2014; Roy et al., 2017). Some data providers prohibited provision of interactive maps at field scale resolution because they perceived locations of outbreaks could constitute personal information. Regulatory agencies do have an obligation to protect personal information but detailed spatial data associated with pest and disease outbreaks is not always considered to be personal information (Scottish Information Commissioner, 2022), and therefore, can be released under a UK law that allows any member of the public to request environmental information held by public bodies (Environmental Information Regulations 2004). Greater clarity, cross‐sectoral agreement and staff training of situations where spatial data would constitute personal information is required, as are systems to ensure that genuine personal information can be removed or anonymised from datasets (Scottish Science Advisory Council, 2021). It is also possible in some instances to remove barriers to data sharing using a co‐production approach, depending on the aims of the work and the different relationships between stakeholders (Urquhart et al., 2023). The authors have experience of stakeholders volunteering to make distribution data available during stakeholder workshops where data sensitivities and sharing solutions could be openly discussed. Once made available, there may be a requirement to maintain up‐to‐date distribution data—participants in our survey certainly stressed the importance of this—in which case, monitoring multiple pathogens and automating data workflows to enable periodic releases would make this more cost‐effective (Barwell et al., 2021; Scottish Science Advisory Council, 2021).

It is possible that our survey introduction led participants to believe that the purpose of the decision tree was to risk assess supplementary planting and inflated the ranking of planting as a disease risk factor. However, given 56% of participants stated they were involved in planting and participants had the option to down weight planting as a risk factor, the results do suggest participants were concerned about, or had first‐hand experience of, planting as a disease risk pathway. Concerns about planting differed most between assessors (who were most concerned) and growers (who were least concerned). Assessors may be more informed about cases where disease outbreaks occurred whereas there is no mechanism to growers about the outcomes of locations planted with their stock so information may not flow back to them about disease detections. Stakeholders in favour of planting believed not doing it posed a greater risk to juniper population collapse than *P. austrocedri*. This is highly questionable given the extensive and rapid loss of juniper trees infected with the pathogen (Green et al., 2015) and the low level but observable juniper regeneration found in populations with lower grazing intensity (Broome & Holl, 2017). Lack of clarity over scenarios in which planting is ill‐advised were identified by 12% of participants as a barrier to using the guidelines. The focus on “safe distances” from an outbreak at which to carry out actions such as planting was misplaced and may not be as effective at reducing risks of pathogen introduction and spread as evaluating and reducing site‐specific risk factors or pathways. A summary of these results was communicated back to survey participants via email and also presented to the Juniper Group England, set up to aid information sharing between practitioners involved in creating or managing juniper populations. The results of the stakeholder exercise ranking abiotic and biotic risk factors driving infection of juniper informed the selection of variables for a national *P. austrocedri* risk model that aims to provide greater clarity of disease drivers at landscape scale (F. Donald, unpublished data). A simple two‐sided flyer specifically addressing risks of restoration juniper planting was also subsequently co‐developed with a stakeholder group as a result of this survey (Green, 2022). Decision tools are only useful; however, where a range of management options exist and may cease to be useable if a consensus is reached that the risk of juniper planting outweighs the intended benefits.

In keeping with findings by Dunn and Laing (2017), survey participants were more pre‐occupied with the relevance of the decision tools than their credibility or legitimacy. However, a small group of participants felt the decision tree overrode their own experience. One stakeholder also thought the guidelines lacked *comprehensiveness* with regard to statutory requirements for juniper management on designated sites. Responses were obtained from a wide range of stakeholders involved in juniper management but did under‐represent views from landscapers, larger commercial growers for whom juniper is a small component of their overall business, agricultural (compared with forestry) agents and all stakeholders based in Northern Ireland. The responses also represent a single timepoint within a fluid stakeholder landscape where additional sectors may, in future, play a larger role (e.g. agriculture, perhaps influenced by revised agri‐environment schemes post‐EU exit). The survey was disseminated during workplace disruption caused by the COVID‐19 pandemic and it is unclear how this impacted participation (e.g. if some sectors were under‐represented because of furlough). Respondents may have been reticent to respond to the survey if e‐mailed by an author they didn't know, and responses about the interactive maps may have been influenced by knowing that F. Donald designed them and would receive their unanoymised feedback. However, the wording and flow of the questions was carefully considered to maintain neutrality across the survey and open‐ended questions were used to afford respondents the space to justify their views (Supplementary Information S1) so the qualitative data collected should be largely unaffected by these factors.

**FIGURE 3:**
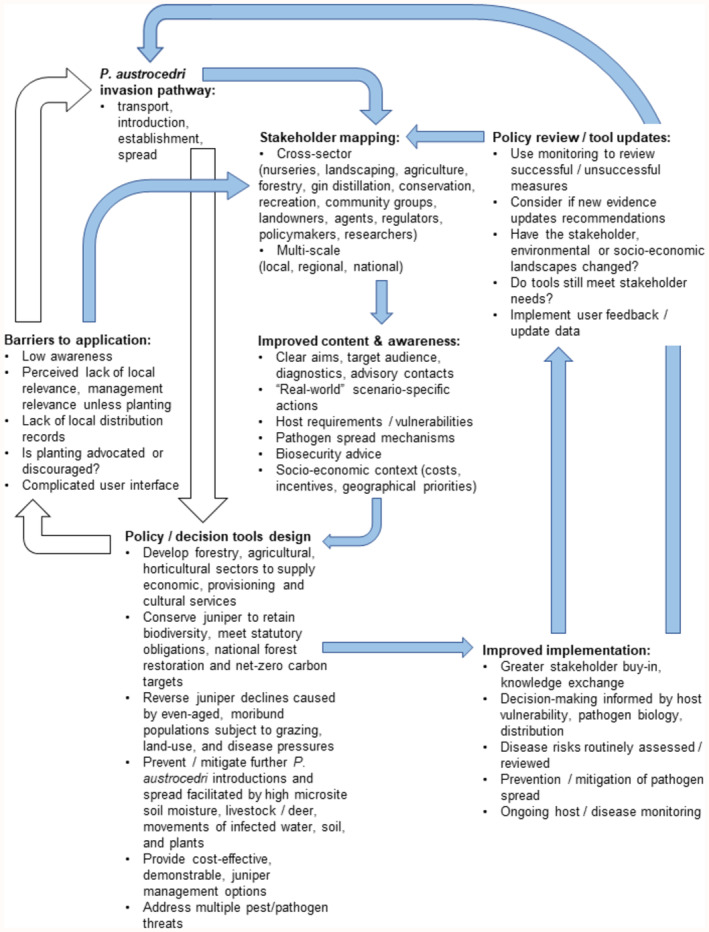
Flowchart outlining the potential benefits associated with co‐producing policy/decision tools with stakeholders using specific examples from *P. austrocedri* infection of wider environment juniper populations. Designing policy without stakeholder engagement (white arrows) may lead to application barriers and increased plant pathogen risks compared with co‐producing policies/decision tools (blue arrows) to identify barriers and improve content, awareness, and implementation. Engagement may be iterative requiring several reviews and may reduce rates of infection if not prevent new disease outbreaks (Colquhoun & Kerp, 2007; Creissen et al., 2019).

We used the information from the survey to design a flowchart (Figure 3) outlining the potential benefits of co‐producing decision tools with stakeholders, the principles of which apply to any plant health management strategy. These principles include conscientious stakeholder mapping to ensure all stakeholders along the invasion pathway are considered. Stakeholder engagement can be expensive and time‐consuming and it is unrealistic to expect government agencies and research institutes will have the resources to fully co‐produce every tool or piece of guidance. This then makes stakeholder mapping particularly important because it identifies who needs to be involved in each part of the process (e.g. content design, evaluation, communications), who the work is most applicable to, who will face the most implementation barriers, where biases will exist if stakeholder groups are not included and who will be most influential in sharing the outcome. Communications of new government guidelines have somewhat improved since 2017, for example, better use of interested party websites for signposting, and wider use of blogs and social media (Barbrook, 2024, pers. comm) but using stakeholder knowledge exchange networks remains an effective way to increase awareness and promote the use of decision tools (Breukers et al., 2009; Creissen et al., 2019; Figure 3). Another principle is the need for iterative co‐design, not only to ensure that management strategies evolve alongside new outbreaks or scientific discoveries but also to improve knowledge exchange between stakeholder groups. For example, growers can learn from land managers about how well nursery supplied material fares following planting, or policymakers could hear which guidelines agents never recommend because the wording is unclear or there is insufficient accessible data. Gathering and responding to such feedback is required to ensure decision tools successfully prevent new pest or disease outbreaks and reduce spread (Figure 3). Examples of successful iterative co‐design include the production of toolkits to help landowners manage ash dieback in Scotland (The Tree Council, 2021) and testing realistic management scenarios to control forecasted spread of *Phytophthora ramorum* in the United States of America with the latter involving the research sector in knowledge co‐production (Jones et al., 2021).

## AUTHOR CONTRIBUTIONS

**Flora Donald:** Conceptualization (lead); formal analysis (equal); investigation (equal); methodology (equal); project administration (lead); resources (equal); visualization (lead); writing – original draft (lead); writing – review and editing (equal). **Carrie Hedges:** Conceptualization (supporting); formal analysis (equal); investigation (equal); writing – review and editing (equal). **Bethan V. Purse:** Investigation (supporting); methodology (equal); supervision (lead); writing – review and editing (equal). **Nik J. Cunniffe:** Formal analysis (supporting); methodology (equal); supervision (supporting); writing – review and editing (equal). **Sarah Green:** Investigation (supporting); supervision (supporting). **Festus A. Asaaga:** Formal analysis (supporting); investigation (supporting); methodology (equal); writing – review and editing (equal).

## FUNDING INFORMATION

F. Donald was funded by the Scottish Forestry Trust, Scottish Forestry, Forest Research, NatureScot, the Royal Botanic Garden Edinburgh and the UK Centre for Ecology and Hydrology. C. Hedges was funded by Cumbria Woodlands. B. V. Purse was additionally supported by the NERC NewLEAF project, NE/V019813/1, and the Scottish Plant Health Centre (Project code: PHC2019/06).

## CONFLICT OF INTEREST STATEMENT

The authors have no conflicts of interest to declare.

## Supporting information


Data S1

## Data Availability

A version of the dataset with all direct and indirect personal identifiers removed has been uploaded to the Dryad repository DOI: 10.5061/dryad.msbcc2g4n and will be made publicly available if the manuscript proceeds to publication.
