# Supplementary material for: Utility of decision tools for assessing plant health risks from management strategies in natural environments
Source: Ecol Evol. 2024 May 2;14(5):e11308. doi: 10.1002/ece3.11308 (PMC11066480; doi:10.1002/ece3.11308)
Supplement: Supplementary file 1 — Data S1 [file ECE3-14-e11308-s001.docx]

# Supporting Information

## Supplement S1 Reproduction of the self-completion stakeholder survey


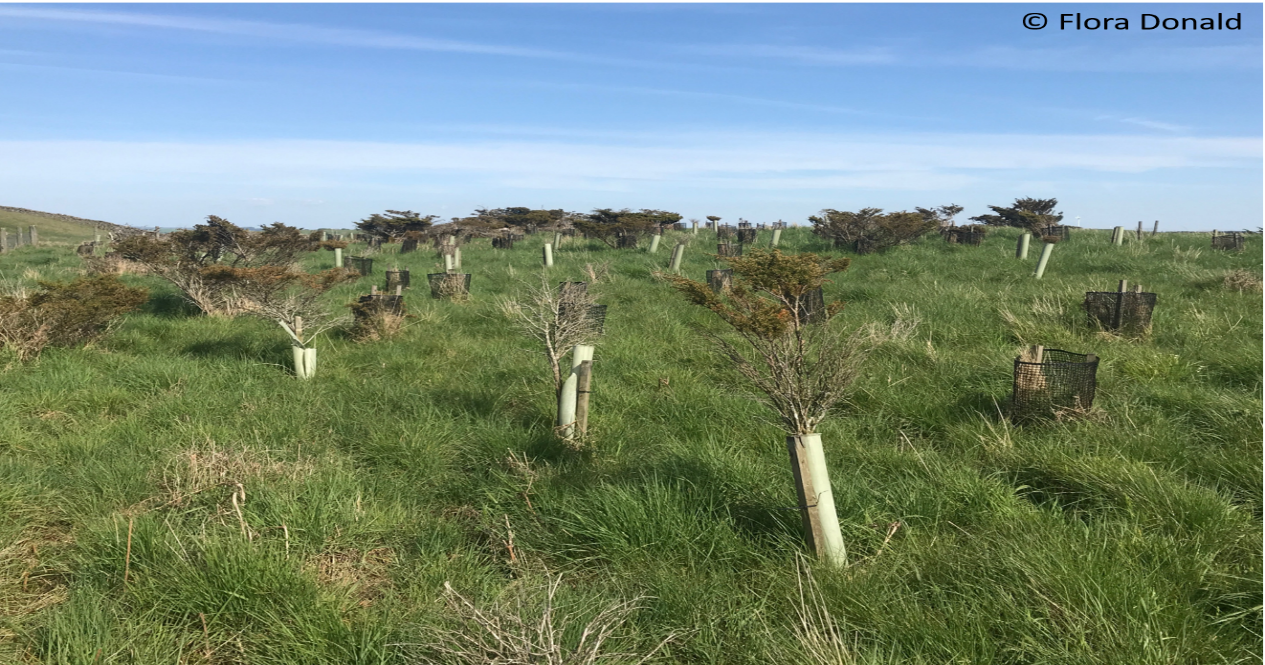


**Risk assessing supplementary juniper planting**

Thank you for taking time to complete this questionnaire. The purpose of this questionnaire is to understand the extent to which the decision tree in the juniper management guidance is currently used and identify the need for additional tools to aid risk assessment of juniper populations in relation to the plant pathogen *Phytophthora austrocedri*. The overarching aim of our research is to identify risk factors for *P. austrocedri* infection of UK juniper populations to inform management strategies for juniper conservation.
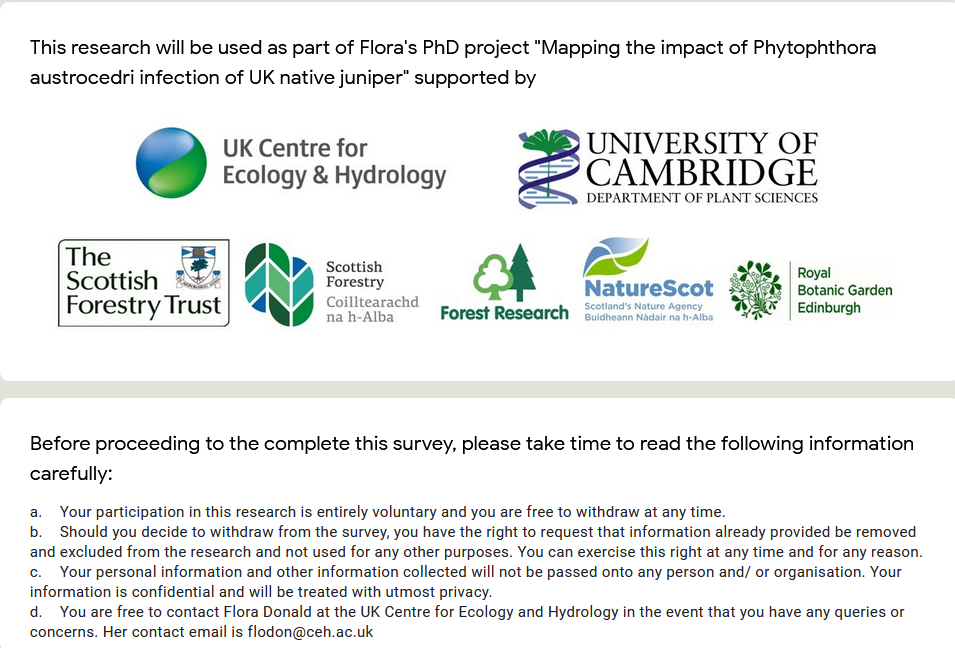


Are you willing to proceed with this survey? Tick here to confirm consent: ☐Yes ☐No

1. Could you tell us about your experience and role?

2. Does your role include management of juniper or *P. austrocedri*? ☐Yes ☐No

3. Are you involved in juniper planting? ☐Yes ☐No

**Juniper management guidance accessibility**


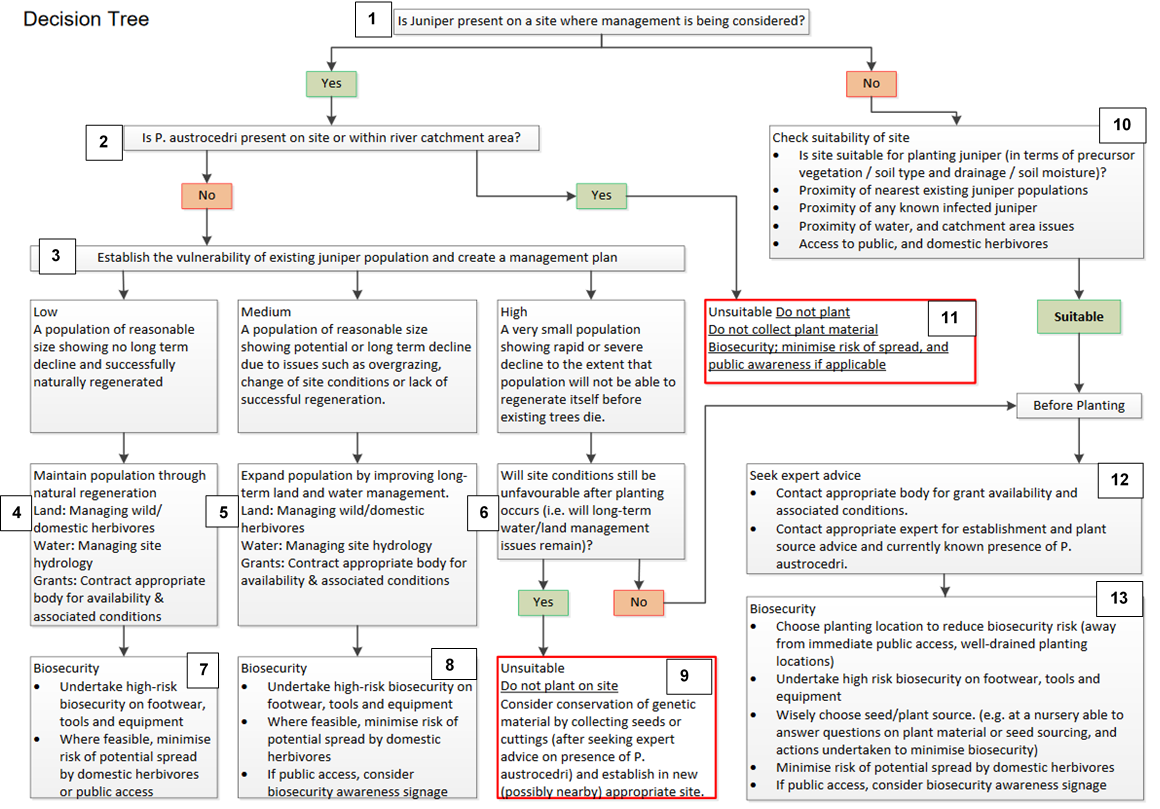
Juniper has widely been planted for conservation purposes to re-invigorate dwindling, native populations. However, the introduced plant pathogen, *Phytophthora austrocedri*, is now causing widespread mortality in juniper populations across the UK. Supplementary juniper planting is a potential pathway by which the pathogen could be introduced or spread. Management guidance for juniper was issued by DEFRA in 2017, including a decision tree to help assess the need and site suitability for supplementary planting. We aim to find out how the decision tree could be made more accessible to inform juniper conservation strategies.

4. Do you already use the decision tree in your work? ☐Yes ☐No

5. If Yes to Q.4, tell us how you have used it?

6. If No to Q.4, please explain why.

7. How likely are you to use the decision tree to assess the suitability of planting juniper at a proposed location? ☐Very likely ☐Likely ☐Unsure ☐Unlikely ☐Not at all likely

8. In its current presentation, which parts of the decision tree:

a) are additional to any planting decision process or risk assessment you currently use?

b) are most useful for determining the suitability of planting? Why do you say that?

c) would you find difficult to assess and why?

9. Do you have any additional comments about the decision tree?


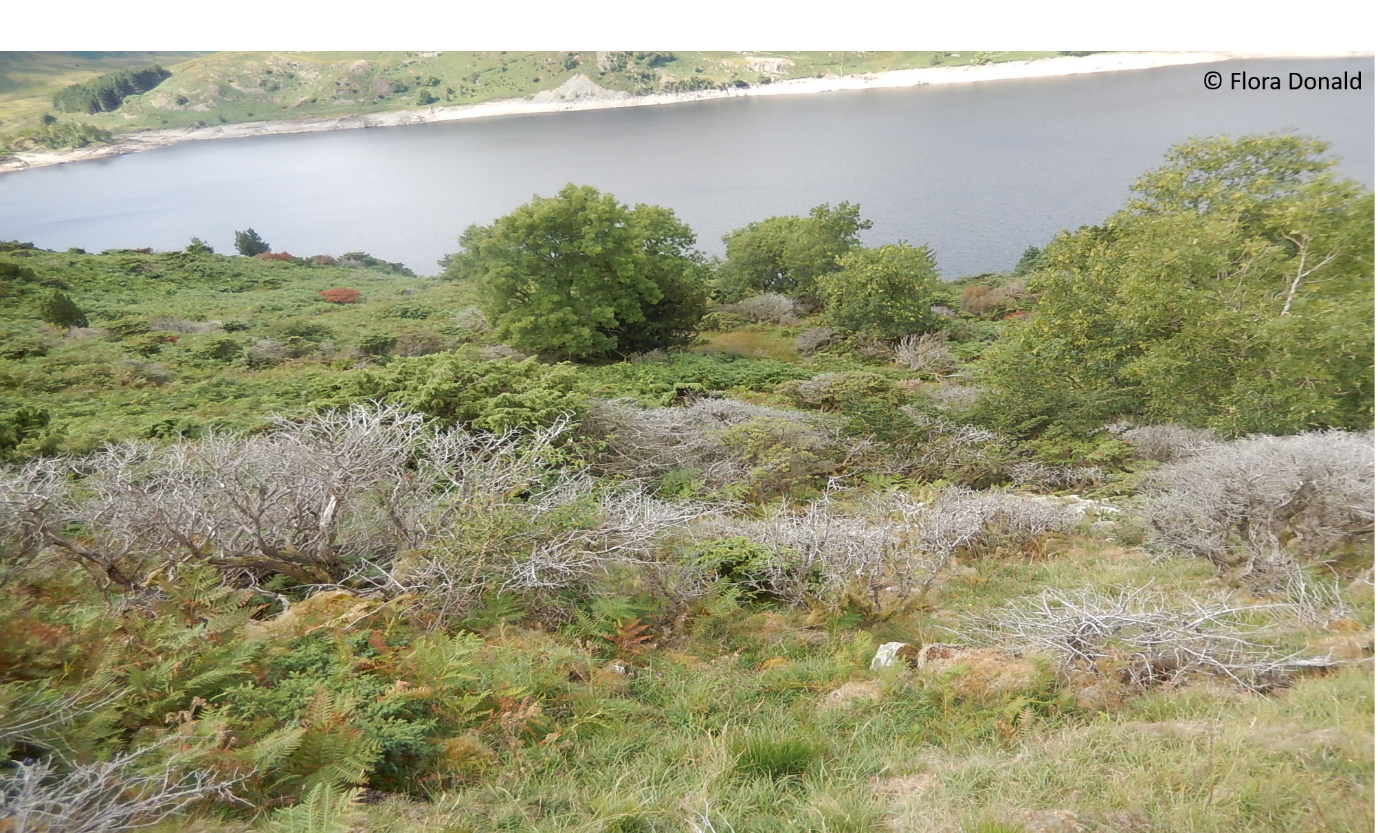


**Maps of *P. austrocedri*, native juniper and planted juniper**

We are developing a range of national level and sub-national level maps (a) showing where *P. austrocedri* has already been recorded in relation to native juniper and planted juniper and (b) predicting where else in the UK landscape conditions are suitable for *P. austrocedri* to establish and spread.

Access example maps here: <https://floradonald-juniper-planting-2020.shinyapps.io/Planting2/>

10. Do you already use maps of juniper and/or *P. austrocedri* for management decision-making? ☐Yes ☐No

11. If yes, how do you use them?

12. If yes, where are these maps sourced from?

13. If a map of *P. austrocedri* were made available, would you be more or less likely to conduct a site visit to check for the pathogen? ☐Very likely ☐Likely ☐Unsure ☐Unlikely ☐Not at all likely

14. Please may you explain your response?

15. Which scale of geographical information would be most useful to you? (Tick as appropriate) ☐National ☐Sub-national ☐Other (please specify)

16. Why do you say this?


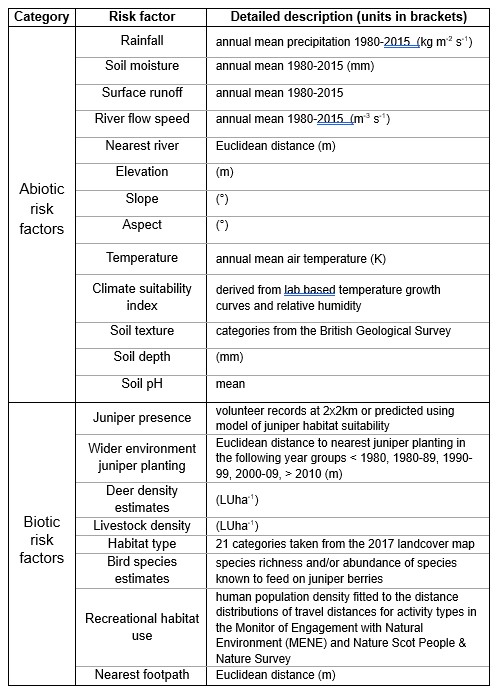
To understand the national distribution of *P. austrocedri*, our model may include some of the following risk factors:

17. Which of the abiotic risk factors do you think are most likely to promote infection of juniper? Please pick your top five risks where 1 is most important. (Please mark one column per row)

|  | **Rainfall** | **Soil moisture** | **Surface runoff** | **River flow** | **Nearest river** | **Elevation** | **Slope** | **Aspect** | **Temperature** | **Climate suitability** | **Soil texture** | **Soil depth** | **Soil pH** |
| --- | --- | --- | --- | --- | --- | --- | --- | --- | --- | --- | --- | --- | --- |
| 1 |  |  |  |  |  |  |  |  |  |  |  |  |  |
| 2 |  |  |  |  |  |  |  |  |  |  |  |  |  |
| 3 |  |  |  |  |  |  |  |  |  |  |  |  |  |
| 4 |  |  |  |  |  |  |  |  |  |  |  |  |  |
| 5 |  |  |  |  |  |  |  |  |  |  |  |  |  |

18. Which of the biotic risk factors do you think are most likely to promote infection of juniper? Please pick your top five risks where 1 is most important. (Please mark one column per row)

|  | **Juniper presence** | **Juniper planting** | **Deer density** | **Livestock density** | **Habitat type** | **Bird species** | **Recreation** | **Nearest footpath** |
| --- | --- | --- | --- | --- | --- | --- | --- | --- |
| 1 |  |  |  |  |  |  |  |  |
| 2 |  |  |  |  |  |  |  |  |
| 3 |  |  |  |  |  |  |  |  |
| 4 |  |  |  |  |  |  |  |  |
| 5 |  |  |  |  |  |  |  |  |

19. Are there any additional factors you think may be driving the spread of the pathogen that we should explore?

20. In your view, how could we keep the juniper planting map (see <https://floradonald-juniper-planting-2020.shinyapps.io/Planting2/> ) up to date?

21. What do you see as the benefits of keeping the planting map up to date, if any, to your management?

**Personal information**

Please tell us a little more about your work with juniper.

Your name:

Job title:

Organisation:

Role description or specialisation:

Geographical area (e.g. UK wide, national, county):

Email address:

Would you be interested in participating in a follow-up workshop to discuss the tools we develop? ☐Yes ☐No

**Many thanks for participating in our research.**

**We expect to share the model results with you by March 2021.**

**
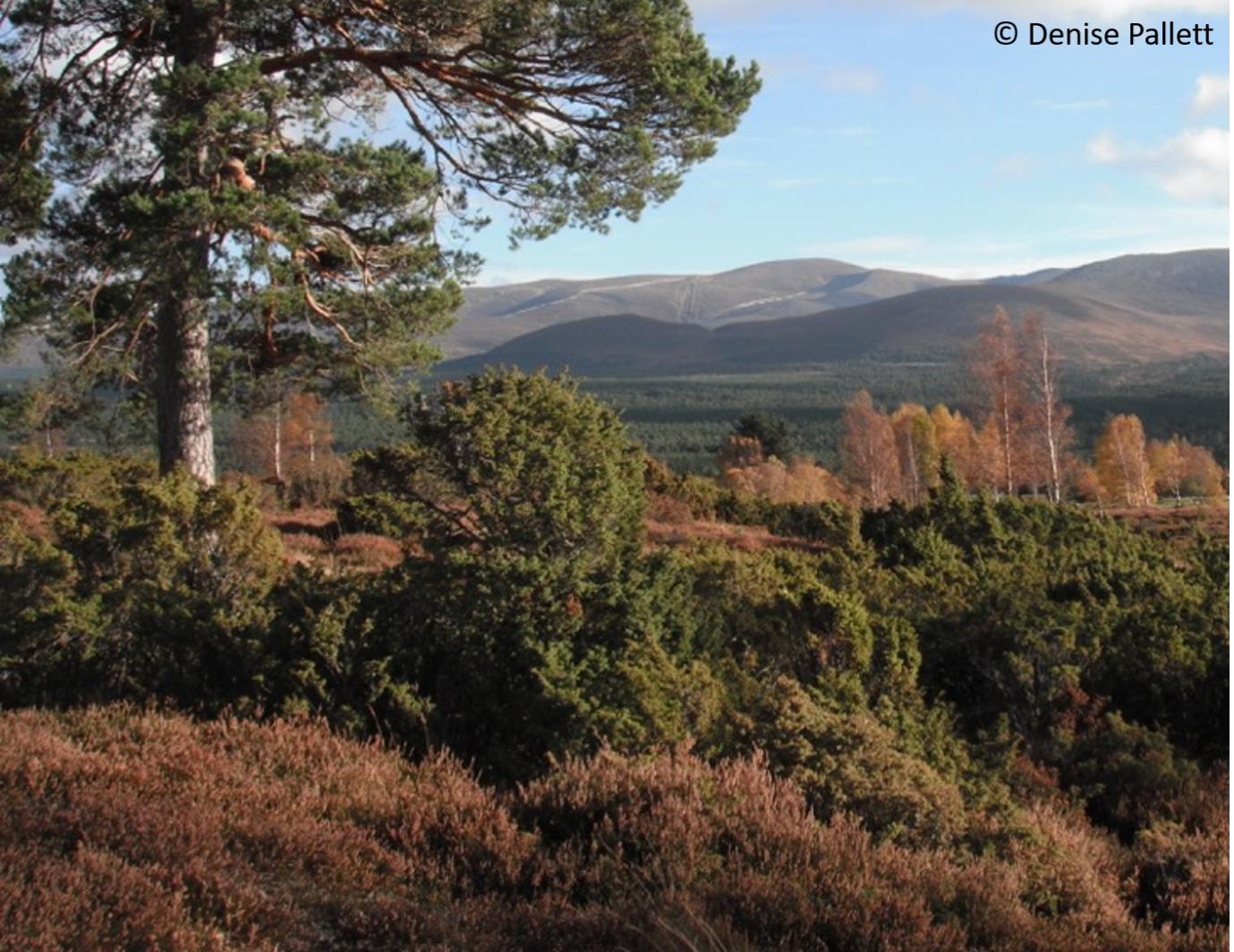
**

## Supplement S2 Reproduction of the ‘juniper planting map’

Question 20 of the survey directed participants via a hyperlink to a Shiny app developed by the authors presenting interactive maps of *P. austrocedri* and native juniper distributions (top) overlaid with known planting locations shown by year (bottom). The maps were derived using the methods described in the main text (see section 2.3 Survey Design). The app text and static captures of the maps are reproduced below.

**UK incidence of *Phytophthora austrocedri* infecting native juniper**

Records of *P. austrocedri* are positive qPCR results collated by Forest Research, Forestry Commission and FERA displayed at 1km resolution, labelled with the year of first detection.
Blue squares show 2x2km records of *Juniperus communis* s.l. observed 1990-2020 by the Botanical Society of Britain and Ireland filtered to exclude records notated as alien or planted.


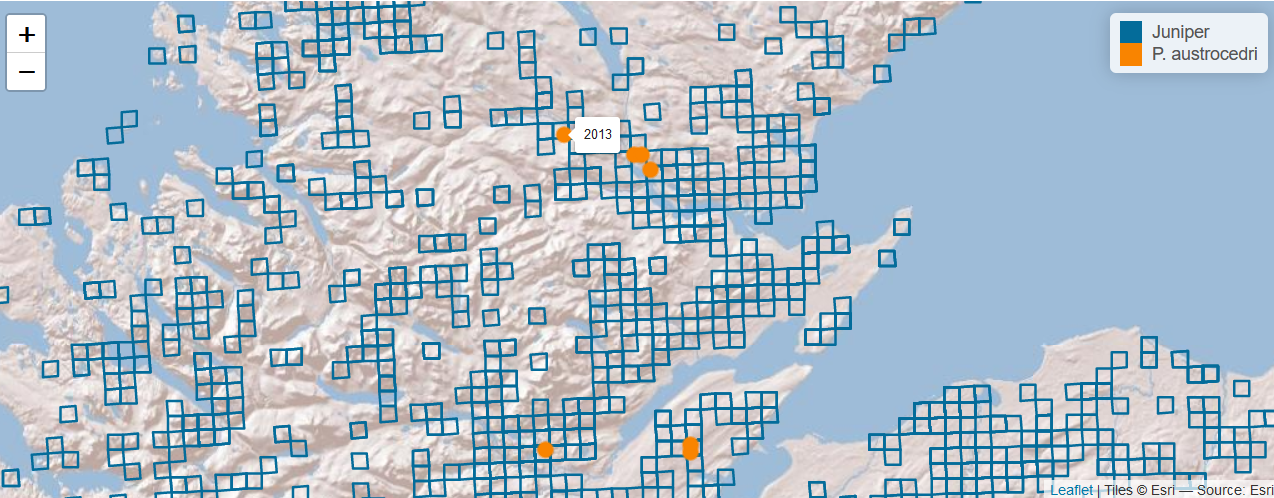


**Juniper planting in the wider environment**


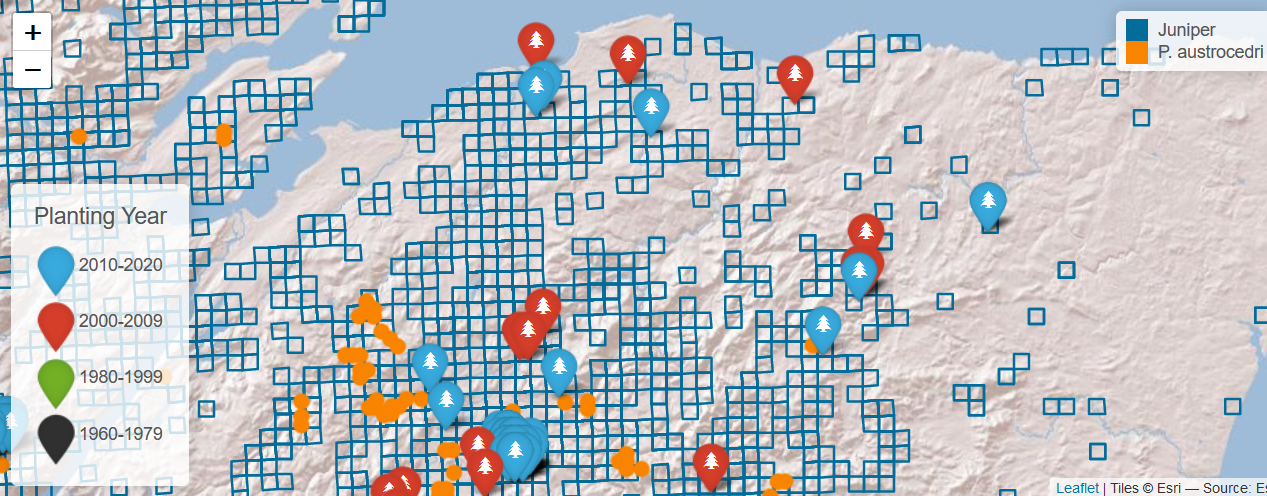
Records of planted juniper compiled with kind permission from individual land managers.
Juniper planting locations are shown according to year of planting (colour categories) and displayed at 2x2km resolution. We are uncertain how long foliage symptoms take to develop following infection with *Phytophthora austrocedri* but expect populations planted after 2009 may still be asymptomatic.

##

## Supplement S3 Additional stakeholder survey information supplementing main results

### **Participant descriptions of their role**

Question one of the survey asked participants to explain their experience and role. Keyword analysis of these responses resulted in identification of twelve activities mentioned by stakeholders as connected with their role (Table S1). Three quarters of these activities were carried out by more than one stakeholder type (Table S1). Question two was used to calculate the proportion of participants conducting each activity who identified as users and non-users of the decision tree in the juniper management guidelines (DEFRA, 2017) (Figure 1).

Table S1. Activities described by survey participants as connected with their roles, mapped to stakeholder type. The activity name relates to that displayed on Figure 1.

| **Activity** | **Description** | **Stakeholder type(s)** | | | |
| --- | --- | --- | --- | --- | --- |
|  |  | **Manager** | **Agent** | **Assessor** | **Grower** |
| Planting | Is or has been involved with planting juniper in the wider environment | x | x |  | x |
| Raise stock | Grows juniper either commercially or non-commercially | x | x |  | x |
| Extant juniper | Manages juniper populations in the wider environment | x | x |  |  |
| Advice | Provides advice about managing existing or creating new juniper populations |  | x | x |  |
| Collect material | Collects juniper for propagation or flavourings / culinary purposes |  |  |  | x |
| Restoration | Restores existing woodlands | x | x |  |  |
| Woodland creation | Creates areas of new woodland | x | x |  |  |
| Monitoring | Monitors protected species or habitats including population fitness assessments |  | x | x |  |
| Surveillance | Conducts surveillance for pests and diseases |  | x | x |  |
| Ex sit-conservation | Manages juniper populations for conservation purposes in an alternative location e.g. a botanic garden |  |  |  | x |
| Outreach | Communicates with a range of stakeholders for the purposes of environmental education |  | x |  |  |
| Planting advice | Provides advice specifically about planting juniper in the wider environment |  |  | x |  |

### **Participant decision tool preferences**

Responses from survey participants that identified uses for the decision tree and/or distribution maps were explored to find out if tool preferences varied with stakeholder type. Two of 41 participants did not identify a use for either the decision tree or the interactive maps. Most of the other participants (61%) detailed uses for both decision tools (Table S2, Figure S1). Although the differences in decision tool preference were not statistically significant, all but one assessor specified uses within their role for both the decision tree and maps (89%). There was less consensus among growers who described uses for either the decision tree or the maps (Table S2, Figure S1).

*
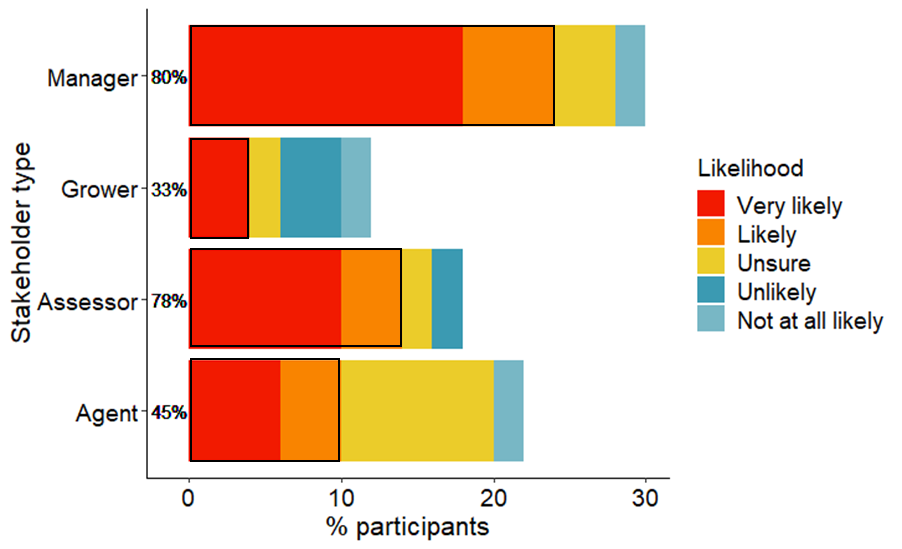
*Table S2. Number and percentage (in brackets) of participants per stakeholder type who described uses for the decision tree and/or distribution maps, or no uses for either tool, in relation to their work. Column totals report the total number (n=41) and percentage of all participants using each decision tool category.

| **Stakeholder type** | **Decision tree & maps** | **Maps only** | **Decision tree only** | **Neither tool** |
| --- | --- | --- | --- | --- |
| Agent | 6 (55%) | 4 (36%) | 0 (0%) | 1 (9%) |
| Assessor | 8 (89%) | 0 (0%) | 1 (11%) | 0 (0%) |
| Grower | 1 (17%) | 2 (33%) | 2 (33%) | 1 (17%) |
| Manager | 10 (67%) | 3 (20%) | 2 (13%) | 0 (0%) |
| Total | 25 (61%) | 9 (22%) | 5 (12%) | 2 (5%) |

Figure S1. Likelihood attributed to use of the decision tree and maps to assess suitability of future juniper planting projects. The percentage of participants (n=41) that selected each likelihood category are shown according to stakeholder type, and the percentage within each type likely or very likely to use tree (shown in red/orange) are reported right of the y-axis.

Thematic analysis of survey responses to questions about the most useful parts of the decision tree, interactive maps and reasons to keep the maps up to date revealed six or seven sub-themes for each category (Table S3).

Table S3. Themes identified from survey responses (n=41) that addressed the utility of the decision tools examined i.e. the decision tree and the interactive maps of *P. austrocedri*, native and planted juniper distributions showing the number (n) and percentage (in brackets) of survey participants whose responses addressed sub-themes.

| **Theme** | **Sub-theme** | **n** |
| --- | --- | --- |
| Most useful parts of the decision tree | all of it | 9 (22%) |
|  | site suitability checklist | 7 (17%) |
|  | site vulnerability checklist | 5 (12%) |
|  | as a tree for risk assessment | 3 (7%) |
|  | assess need for planting | 2 (5%) |
|  | assess longevity of planting | 1 (2%) |
| Uses for interactive maps | assess infection risk | 11 (27%) |
|  | inform management decisions | 6 (15%) |
|  | assess planting suitability | 5 (12%) |
|  | sourcing decisions | 4 (10%) |
|  | early disease detection | 1 (2%) |
|  | raise awareness of nursery biosecurity | 1 (2%) |
|  | monitor losses | 1 (2%) |
| Reasons to keep maps updated | plan management | 14 (34%) |
|  | contain disease | 5 (12%) |
|  | risk assessments | 4 (10%) |
|  | track sources of infection / target monitoring | 4 (10%) |
|  | evidence for funding applications | 1 (2%) |
|  | save clients' money | 1 (2%) |

### **Spatial scale preferences for host/pathogen maps**

Survey questions 15 and 16 asked participants to specify the spatial scales at which maps of *P. austrocedri*, juniper and juniper planting would deliver maximum benefit to their role connected with juniper management (Supplement S1). Local maps were most popular with requests for detailed (6-8 figure) grid references and the ability to zoom in further than allowed on the 1-2km resolution interactive map provided with the survey (Table S4). Twelve percent of participants who worked across the UK requested national (UK-wide) distribution maps but a further 27% requested national maps to contextualise local maps (Table S4). Maps displaying pathogen and juniper distributions within single counties or countries within the UK were requested by 20% of participants and only one participant identified the need to contextualise their region within the wider UK distribution (Table S4).

Table S4. Number (n) and percentage (in brackets) of participants who chose each spatial scale as the most useful for distribution maps to aid decision-making. An example quote representing the most frequent justification for using maps at each scale is provided.

| **Spatial scale** | **n** | **Exemplar quote** |
| --- | --- | --- |
| Local (sub-county) | 16 (39%) | “*I'd want site grid refs or a zoomable map to work out exact locations. Your maps need place names and roads or some way of working out where the squares actually are e.g. national grid.”* (Manager 6) |
| Regional (country / county) | 8 (20%) | *“we operate on a regional and countywide basis.”* (Assessor 7) |
| National (UK) | 5 (12%) | *“We could be asked to propagate from other areas in the future.”* (Grower 3)  *“I have various clients around the country and the more information the better.”* (Manager 3) |
| Local & national | 11 (27%) | *“A national overview is important so that regional/local decisions can be put into the wider context.”* (Agent 6) |
| Regional & national | 1 (2%) | *“I work on a regional scale but it is good to understand the national picture too.”* (Manager 11) |

### **Participant recommendations to improve decision tool accessibility**

Survey question 12 asked participants to detail where they sourced information if they already use maps of juniper and/or *P. austrocedri* for management decision-making (Supplement S2). Private sources of information including organisation specific or participant’s own knowledge were reported most frequently (24% participants) (Figure S2a). Publicly accessible sources were cited by 15% of participants; the Forest Research website was used most often (7%) followed by individual participants (2% each) who cited using the National Biodiversity Network (NBN) gateway (The National Biodiversity Network, 2021), the Ecological Site Classification Decision Support System (ESC) hosted on the Forest Research website that matches site factors with ecological requirements of different tree species and woodland communities to aid site-based choices about suitable plantings (Forest Research, 2021), and “FC Tools” more generally which could correspond to ESC or another platform (for example the Forestry Commission Land Information Search (FC LIS) (Forestry Commission, 2021)) (Figure S2a). Both the ESC and NBN currently only host records of juniper, and not *P. austrocedri*, as is the case for records accessed by a further 9% of participants from maps hosted by the Botanical Society of Britain and Ireland (BSBI) or published by Plantlife (Ward and Shellswell, 2017) (Figure S2a).

Participants suggested a variety of information sources that could be used to improve the distribution maps and maintain up to date records but the question posed was insufficiently specific to always pinpoint if answers related specifically to juniper planting locations, those infected with *P. austrocedri*, or both (Figure S2c). Thirteen percent of participants suggested actively writing to public bodies (7%), nurseries (2%) or individual stakeholders (2%) to request records or obtain information from management plans (2%):

“Regular updates from FLS/FE [Forest and Land Scotland / Forestry England] sub-compartment database, updates from forestry grant scheme payment every 6 months. Not sure this would capture all the charity sector planting or enrichment planting schemes though. Agreements for updates could be made with main players though.” (Manager 11),

while a further 4% suggested liaison with pathologists or forest reproductive material officers (FRMOs) who:

“must be notified of any marketing of juniper including all seed collecting and plant supply” (Grower 4).

Unsurprisingly, when asked where the decision tools could be hosted to improve awareness and accessibility, many of the suggestions included the platforms already cited as current sources of information, such as the Forest Research website (5%), NBN (2%) and Plantlife (2%) (Figure S2b). Additional suggestions included the popular citizen science platform iNaturalist (2%), a bespoke central repository specifically for juniper (2%), an online juniper management forum (2%) or incorporation into existing governmental land management mapping software including FC LIS (7%) and the DEFRA MAGIC viewer (5%) (Figure S2b). Except for the NBN and iNaturalist, however, these platforms only permit data entry or modification by internal staff members. A large proportion of survey participants suggested data entry should be performed by the data handlers themselves, ideally entering information directly via a web interface or app (15%), compared to making public agencies (2%), action groups (2%) or a dedicated operative (2%) responsible to update the distribution maps (Figure S2d). Further collaboration between these platforms and stakeholders is required, therefore, to add functionality for data entry to the existing platforms or explore further options.


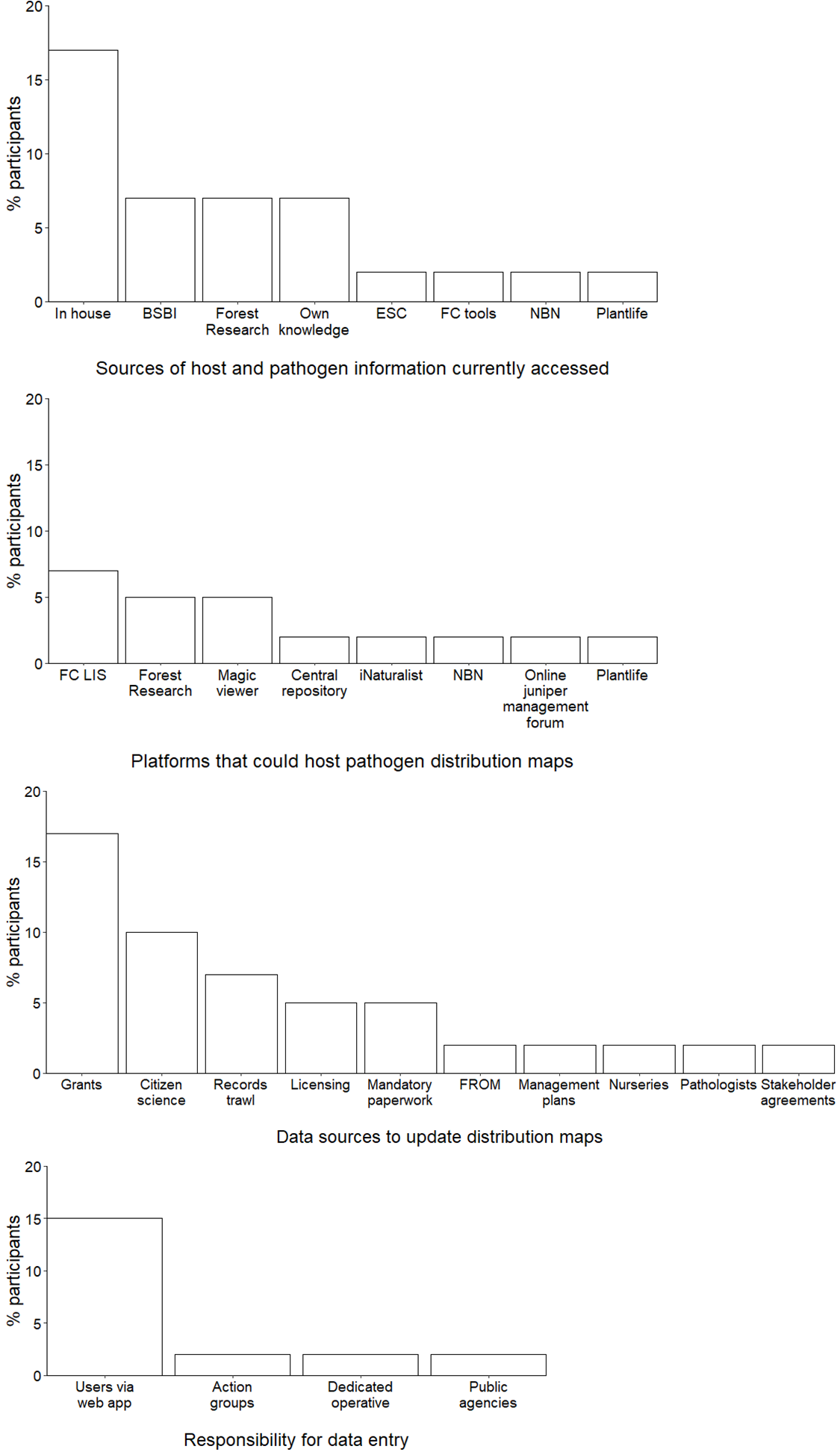


**a**

**b**

**c**

**d**

Figure S2. Percentage of participants (n=41) a) identifying sources of juniper and *P. austrocedri* information they currently access, b) platforms where the new maps could be hosted, c) sources that could be used to update the interactive distribution maps and d) who could be responsible for ongoing data input and map maintenance.

### **Perceived risks of juniper planting**

Question 18 in the survey (Supplement S1) asked participants to pick five out of a possible eight biotic risk factors they perceived as most likely to drive *P. austrocedri* infection of juniper and to rank them in order of importance (Figure S3). Assessors appeared most, and growers least, concerned about planting (89% and 30% ranked planting 4 or 5 respectively) (Figure S3) but the differences were not statistically significant between stakeholder types (Holm-Bonferroni corrected p=0.25).

***
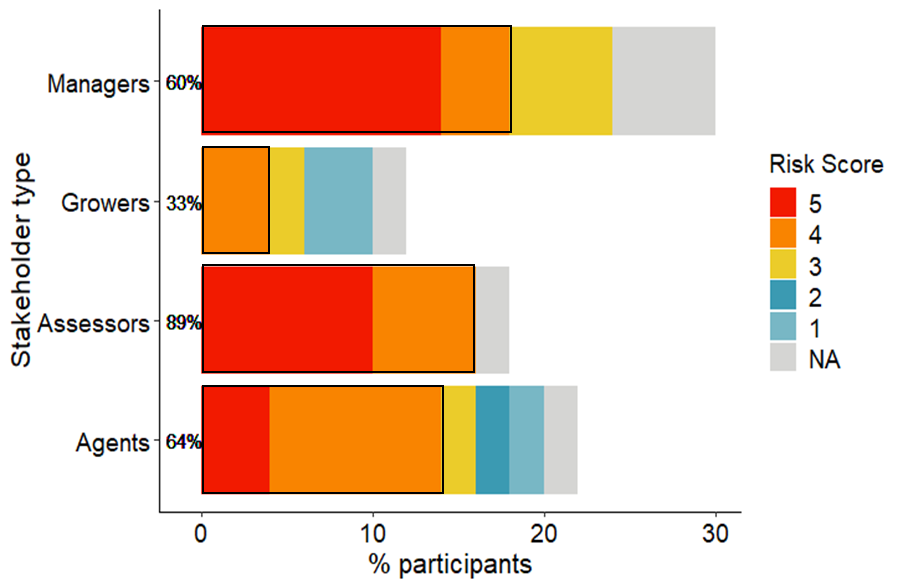
***

**Figure S3.** Percentage of participants (n=41) attributing each risk score to juniper planting according to stakeholder type. The percentage of participants per stakeholder type who ranked planting as a highly important risk factor for *P. austrocedri* (risk score 4 or 5) is written at the right of the y-axis.

REFERENCE:

Forest Research. (2021). Ecological Site Classification Decision Support System. <http://www.forestdss.org.uk/geoforestdss/>

The National Biodiversity Network . (2021). NBN Atlas . https://nbn.org.uk/
